# More beneath the surface? Root *versus* shoot antifungal plant defenses

**DOI:** 10.3389/fpls.2013.00256

**Published:** 2013-07-12

**Authors:** Dirk Balmer, Brigitte Mauch-Mani

**Affiliations:** Institute of Biology, University of NeuchâtelNeuchâtel, Switzerland

## Introduction

Let's get dirty! Plant roots are deeply embedded in clammy soil, and working with them means digging into the mud, hoping not to crush the fragile root branches and flimsy root hairs. In short, working with roots can be a tedious activity. This might partly explain why, over decades, roots could maintain a subsistence fairly hidden from the scrutinizing eyes of plant researchers—until the past few years. Plant breeding has been mainly driven by the search for aboveground traits; as a consequence, roots are now even claimed to be a potential key for a second green revolution (Gewin, 2010). Promising studies aiming to improve crop yields focus on root architecture and the genetics underlying root development. For instance, wheat lines exhibiting deeper and fast-growing roots were isolated and crossed with commercial cultivars, in the hope of generating drought-tolerant lines (Kirkegaard et al., 2007). Similarly, drought-stressed maize lines were shown to have a higher yield when specifically adapting their root structure by building an intercellular air space compared to maize varieties that were not able to adapt their root architecture (Zhu et al., 2010). As if handling abiotic stress would not be already a substantial task, roots are also garrisoned at the frontline against pathogens and pests. Soil-borne pathogens such as *Fusarium*, *Pythium*, or *Phytophthora* are infamous for being vicious parasites that can devastate entire harvests (Okubara and Paulitz, 2005). However, the lack of a convenient accessibility of root systems renders the task of controlling such pathogens more than challenging. Consequently, the knowledge about belowground defense responses is limited, in contrast to well-dissected aboveground mechanisms (Rasmann and Agrawal, 2008). Moreover, studies comparing root and shoot immune responses are scarce. In animal model systems, organ-specific antifungal immune responses are a well-known phenomenon (Lionakis et al., 2011). In plant-microbe models, pathological studies were historically mainly focused on either roots or shoots. Recent studies highlight an organ-specific immunity against fungal pathogens, pointing toward the fact that plants employ a “defense in depth,” a multi-layered organ-specific local and systemic defense system. Elucidating such a defense in depth for given pathosystems will be an important task for future studies in plant pathology.

## Organ-specificity: the paradigm of being different

Plants and their pathogens have undergone an intimate co-evolution, culminating in intricate plant-pathogen interaction patterns where pathogens can have a narrow or wide host range. The evolutionary paradigm of host specialization holds also true for organ-specific infection processes. Organ-specificity is well described for plant pathogen interactions and usually designated by characteristic disease symptoms such as root and stem roots, stem cankers, leaf blights, or fruit spots. Intriguingly, it remains largely elusive why some pathogens prefer a given organ, whereas others are able to infect the entire plant. Are these organs so different from each other, and do they possibly employ distinct defense mechanisms? Early work using the *Arabidopsis-Hyaloperonospora arabidopsidis* pathosystem positively affirmed this questioning (Hermanns et al., 2003). In Arabidopsis, resistance against *H. arabidopsidis* is mediated by *R* (resistance) genes, which are expressed in both shoots and roots. Intriguingly, roots were susceptible to various *H. arabidopsidis* strains independent of the presence of *R*-genes, indicating that both organs utilize a distinct defense machinery which is independent of *R*-genes (Hermanns et al., 2003). The few studies about belowground plant immunity point toward a pivotal role of root-secreted secondary metabolites in belowground induced resistance responses (Lanoue et al., 2010). Barley roots infected *by Fusarium graminearum* secrete phenolic compounds and t-cinnamic acid (Lanoue et al., 2010). Root exudates from resistant Gladiolus cultivars were shown to be capable of inhibiting spore germination of *F. oxysporum*, whereas exudates from susceptible cultivars were not (Taddei et al., 2002). An extensive survey comparing shoot and root chemical defense corroborated the fact that shoots and roots are executing distinct defense tactics (Rasmann and Agrawal, 2008). Although most of the compounds present in roots are also present in shoots, the constitutive levels are highly divergent. Moreover, the chemical characteristics of induced defense are diverse. For instance, roots treated with salicylic acid (SA) were shown to set up a transcriptional response that is different from the one in SA-treated leaves (Badri et al., 2008). An intriguing point would be to know if local application of SA would trigger a similar response on locally treated leaves compared to leaves from plants that received a root application.

Taken together, these facts endorse that plants employ an unconventional organ-specific defense. Nevertheless, only a handful of studies are puzzling out organ-specific plant-pathogen interactions by simultaneously comparing shoot and root defenses. For instance, it is known that the typical leaf pathogen *Magnaporthe oryzae* colonizes rice roots in a manner distinct from foliar attacks, and treatment of roots with the chemical resistance inducer benzo-(1,2,3)-thiadiazole-7-carbothioic acid S-methyl ester (BTH) had no protective effect against *M. oryzae*—in contrast to the BTH-treated foliage (Jansen et al., 2006). Intriguinly in this case, it remains elusive if BTH root treatment triggers foliar resistance, as observed in the maize-*Colletotrichum graminicola* pathosystem (Balmer et al., 2013). A similar study reports that roots of the non-host Arabidopsis were generally susceptible to *M. oryzae*, contrary to aboveground parts, which were resistant (Schreiber et al., 2011). Thus, the paradigm of organ-specificity can even be extended to non-host resistance. A comparison of transcriptional responses of *M. oryzae*-infected rice roots and shoots revealed that defense-related transcripts are suppressed in roots, while they accumulate during foliar infection (Marcel et al., 2010). *M. oryzae* also applies organ-specific infection structures which suggest a unique biotrophic lifestyle on roots (Marcel et al., 2010). The mechanisms underlying organ-specific antifungal defense remain largely elusive. A recent study compared above- and belowground defenses of maize against the hemibiotrophic fungus *C. graminicola* (Balmer et al., 2013). This fungus has the staggering ability to infect shoots, roots as well as stalks, thus being a convenient fungal model to study organ-specific defense responses. It has been found that roots respond much faster to fungal infection than leaves, although the disease development is slower on roots (Balmer et al., 2013). In response to *C. graminicola* infection, roots also exhibited higher levels of the defense-associated plant hormones SA, jasmonic acid (JA) and abscisic acid (ABA), and the pathogen-induced metabolomics profile of roots is different from shoots (Balmer et al., 2013). Higher levels of flavonoid compounds that were detected in roots also indicate a higher defensive state of maize roots. Furthermore, *C. graminicola* root infections provoked systemic resistance in the foliage against the same fungus more efficiently than leaf infections, suggesting that roots have an enhanced capability to trigger systemic defense responses in maize (Balmer et al., 2013).

## Looking further below the surface

The current knowledge, albeit being confined, corroborates the fact that shoots and roots utilize a distinct defense machinery. In consequence, two main questions arise that urge to be answered to advance in belowground plant research: Firstly, why are shoot and root defenses distinct to such an extent, and secondly, are roots more (or less) protected against fungal pathogens? Using an evolutionary and ecological approach, organ-specific defense systems could be explained by positive or negative feedback mechanisms between the microbial communities and given plant species. The environment plants are exposed to is highly determinant for the outcome of ecological interactions; comparing soil and air, it becomes obvious that belowground and aboveground plant parts are confronted to completely disparate microbial conditions. The composition of these different conditions likely affects the evolutionary development of organ-specific plant-microbe interactions. For instance, due to the viscosity of the environment, soil microbes are usually highly spatially distributed, thus having likely an effect on the very same plant species on which they accumulate. This could explain why in most of the cases negative effects of soil pathogens are host specific (Bever et al., 2012). Accumulation of host specific pathogens is known to result in negative plant-soil feedback, which consequently favors the growth of non-host plant species. Negative plant-soil feedbacks are also reflected in agriculture by crop rotation techniques, as applied for corn-soybean cultures in the United States, aiming to battle host-specific pathogen aggregation (Bever et al., 2012). In summary, both roots and shoots are subjected to highly distinct plant-microbe community feedback associations, which could explain why roots developed different immune responses.

Dissecting complex soil-born microbe communities has become a more feasible task due to novel high-resolution root microbe profiling methods (Bulgarelli et al., 2012), making apparent that roots are exposed to highly dynamic soil-born microbe communities. Moreover, roots are indispensable for plant survival, thus the plants might likely protect these highly valuable organs by investing in higher constitutive root defenses. In accordance to this hypothesis, crown roots of maize, which are vital for a proper plant development, were shown to contain higher levels of insecticidal compounds compared to other root types (Robert et al., 2012). Hence, it could be concluded that roots bear an advanced defensive state. However, if so, why are roots of the non-host Arabidopsis more susceptible to *M. oryzae* than leaves? Proteomic and metabolomic studies of roots under fungal attack corroborate the fact that roots often have higher levels of antimicrobial compounds. However, these higher levels do not necessarily mean a more efficient induction of defenses. In addition, pathogens are also subjected to an intimate co-evolution with their hosts, and the plant-soil feedback results in a spatial and ecological allocation of soil-born microbes. Fungi that are able to detoxify microbial compounds or even make use of them to target nutrient-rich root tissues are favored in a dynamic root community. Thus, to further study organ-specific plant immunity, it is inevitable to elucidate the dynamics of both plant and pathogens during above- and belowground infection incidents. For a research agenda of plant immunity, analyzing the whole plant and generating a concept of a “defense in depth” is an immediate task. An additional important future quest is the elucidation of systemic defenses upon local shoot and root pathogen attack. Extensive knowledge is available for root interactions with non-pathogenic microbes such as mycorrhizal fungi (Liu et al., 2007) or growth-promoting rhizobacteria (van de Mortel et al., 2012) and especially for herbivores (Soler et al., 2012). It remains barely investigated to which extent leaf tissues are contributing to fungal root infections and *vice versa*. It was proposed that roots can act as dynamic storage organ for defensive compounds (Erb et al., 2009), reasonably manifested by the example of nicotine which is produced in tobacco roots and translocated to the shoot via the xylem (Dawson and Solt, 1959). A similar process would be also conceivable for plant-pathogen interactions, where roots could act as reservoir for antifungal compounds. However, leaf infection of fungal pathogens were recently shown to only trigger minor transcriptional and metabolomic adaptations of roots (Balmer et al., 2013). Hence, it remains highly debatable if there is a root-to-shoot and *vice versa* communication, a phenomenon notably observed in herbivore defense signaling in maize, were aboveground caterpillar feeding induces the belowground accumulation of the cysteine protease Mir1-CP (Luthe et al., 2011).

Roots are a crucial organ, and uncovering belowground defense mechanisms has a great potential being exploited for aboveground plant defense. Therefore, it is time to dig much deeper into belowground plant defense mechanisms, to finally understand better why plant organs are so different, which mechanisms are mediating these differences, and what is the setup of above-belowground and *vice versa* communication during pathogen attack.
